# Pharmacology, physiology and performance: occupational drug use and HIV risk among female entertainment and sex workers in Cambodia

**DOI:** 10.1186/s12954-015-0068-8

**Published:** 2015-10-16

**Authors:** Thomas Crewe Dixon, Song Ngak, Ellen Stein, Adam Carrico, Kimberly Page, Lisa Maher

**Affiliations:** Faculty of Law, University of Sydney, Sydney, Australia; FHI 360, Phnom Penh, Cambodia; University of California, San Francisco, San Francisco, CA USA; University of New Mexico, Albuquerque, NM USA; Kirby Institute for Infection and Immunity, Faculty of Medicine, UNSW Australia, Sydney, Australia

**Keywords:** Amphetamine, Stimulant, Drug, Sex work, Occupation, HIV risk, Cambodia

## Abstract

**Background:**

In Cambodia, HIV prevalence among female entertainment and sex workers (FESW) is up to twenty times higher than in the general population. Use of amphetamine-type stimulants (ATS) has been associated with increased risk of HIV and other sexually transmitted infections in key populations, including FESW. While one in four Cambodian FESW report recent ATS use, little attention has been paid to how the occupational contexts of sex work shape patterns of use. Currently, no HIV prevention interventions target ATS use in this population.

**Methods:**

We conducted in-depth interviews with FESW (*n* = 30) with the goal of exploring experiences and motivations for ATS use and informing the development of a conditional cash transfer (CCT) intervention designed to reduce ATS use and HIV risk. Interviews were conducted and transcribed in Khmer and translated into English. Interview narratives were read and re-read and emerging themes reviewed and refined to develop an initial coding scheme. Data were formally coded using both open and axial coding to clarify and consolidate initial themes.

**Results:**

The most common driver of ATS use among FESW was increased functionality. ATS was seen as a performance enhancer, acting as an appetite suppressant and enabling women to meet the physiological demands of sex work, including long working hours, multiple clients and extended sexual transactions. While our results are consistent with studies linking ATS use to heightened sexual risk, via unprotected and/or prolonged sex, for women in the current study, the negative consequences of ATS use were outweighed by perceived functional benefits.

**Conclusions:**

FESW in Cambodia harness the pharmacological properties of ATS to meet the physiological demands of sex work in a context of limited economic opportunities. There is an urgent need to both provide Cambodian women with options for income generation that do not risk their health and to better regulate the conditions of sex work to provide safer working environments. Structural and economic interventions, including CCT programmes, combined with awareness and enforcement of sex workers’ rights, are also necessary to facilitate harm reduction and occupational health and work safety within the Cambodian sex and entertainment industry.

## Background

Amphetamine-type stimulants (ATS) are synthetic psychostimulants such as methamphetamine, amphetamine and ecstasy which can be injected, smoked or taken orally. Administration results in feelings of euphoria, alertness, increased heart and respiratory rates, blood pressure, perceived increases in confidence and physical strength, and appetite suppression [1]. With prolonged use and in high doses, ATS can produce anxiety, hyper-vigilance, paranoia, psychosis, panic and other adverse effects [1, 2]. Studies also suggest that ATS use can increase libido, lower inhibitions, enhance sexual pleasure, delay orgasm and prolong sexual intercourse [3–7]. ATS use has been associated with increased HIV risk in a range of populations including men who have sex with men (MSM) [4], people who inject drugs [8], non-injection drug users [9], heterosexual men and women [10, 11] and young people [12]. In particular, ATS use is believed to have a disinhibiting effect on sexual decision-making [5] and has been associated with unprotected sex and incident sexually transmitted infections (STI), including HIV [10, 13, 14].

ATS use has increased dramatically throughout Asia in recent years, including in Cambodia [15–17]. The United Nations Office on Drugs and Crime has estimated that there are approximately 500,000 drug users in Cambodia and 30,000 tablets of methamphetamine (yama) are consumed daily [18]. Methamphetamine in pill form is the leading drug of abuse, but crystal methamphetamine use has been rising since 2006 [16, 18]. While approximately 0.8 % of the adult population in Cambodia is estimated to be living with HIV, prevalence is significantly higher among female sex workers, referred to here as female entertainment and sex workers (FESW), with 15–23 % of young women (15–29 years) testing positive in a recent study [14, 19]. Incidence of HIV was 3.6 per 100 person-years and 21.2 per 100 person-years for gonorrhoea and chlamydia. More than one in four (26 %) FESW reported recent ATS use. Factors independently associated with incident STI included duration (per year) of sex work (adjusted hazard ratio (AHR) 1.1; 95 % CI 1.1–1.2) and recent use of yama (AHR 4.3; 95 % CI 1.7–11.0) [14]. Further analyses revealed independent associations between ATS use and higher number of sex partners (adjusted relative ratio 1.49; 95 % CI 1.00–2.21) and incident STI (adjusted odds ratio 5.41; 95 % CI 1.15–25.48) [19].

Related qualitative research found that ATS, primarily yama, was inexpensive, easy to find and commonly used by FESW in Cambodia. Yama was described as a ‘power drug’ (thnam kamlang) which enabled women to work long hours and see more clients. Use of ATS by clients was also common, with some providing drugs for women and/or encouraging their use. Intoxicated clients frequently requested unprotected sex, and strategies typically employed by FESW to negotiate condom use were reported as less effective in this context [20, 21]. Based on these findings, we proposed an implementation science research programme designed to address the intersection between drug use and HIV infection in this setting by providing financial incentives or Conditional Cash Transfers (CCT) [22] to FESW to reduce their use of ATS. As part of the development of this programme, we conducted formative qualitative research to further examine the occupational context of ATS use. Specifically, the current study aimed to clarify gaps in our understanding by documenting patterns of ATS use and the settings in which it occurred, exploring motivations for, and perceived negative consequences of, ATS use and examining experiences of attempting to abstain from or reduce consumption.

## Methods

The Cambodia Integrated HIV/Drug Prevention Intervention (CIPI) project seeks to test and evaluate an intervention to reduce ATS use and HIV risk among FESW. CIPI was designed to assess the impact of new combination HIV prevention intervention options (drug use prevention and microfinance tools) integrated into an existing programme, FHI 360’s *SMARTgirl* which is the national programme to address HIV prevention among Cambodian FESW. *SMARTgirl* provides a range of education and outreach services, especially referrals to HIV testing, and health services for STI, reproductive health and family planning. It is the most widely disseminated HIV programme for at-risk women in the country, estimated to have contact with over one third of the country’s 35,000 FESW. Using a stepped wedge randomised cluster trial in ten provinces, CIPI will evaluate whether the intervention, consisting of a 16-week programme combining a 12-week CCT programme based on urine toxicology screening for ATS and a 1-month weekly aftercare component, results in larger decreases in HIV risk compared to *SMARTgirl*. Also being evaluated is a microenterprise programme combining financial literacy education and microloan opportunities for ATS-free FESW.

In 2013, we conducted in-depth interviews with FESW (*n* = 30) in five provinces (Phnom Penh, Battambang, Banteay Meanchey, Siem Reap and Kandal). Eligibility criteria were that participants be aged at least 18 years, biologically female, understand spoken Khmer, used existing *SMARTgirl* services and reported ATS use and at least two different sexual partners in the last month. Participants engaged in sex work in a range of venues, including brothels, entertainment venues, streets and parks were recruited through outreach by *SMARTgirl* programme staff in each province. Interviews were conducted in Khmer by trained interviewers and took 30–90 minutes to complete. Participants received a US$3 phone card and were provided with transportation assistance.

Interviews were digitally recorded and transcribed verbatim in Khmer. Transcripts were checked for accuracy against the original recordings before being translated into English. Following the general tenets and principles of grounded theory [23], data were analysed using an inductive approach. Interview narratives were read and re-read and emerging themes discussed and refined to develop an initial coding scheme. Data were formally coded using both open and axial coding to clarify and consolidate initial themes [24]. All participants provided voluntary informed consent, and ethical approval for the study was provided by the Cambodian National Ethics Committee and the University of California San Francisco Institutional Review Board. Pseudonyms have been provided so that excerpts from the same participant are easily identified throughout the paper.

## Results and discussion

In order to inform the design of the CCT intervention, we sought to better understand the occupational context and drivers of ATS use in this group. Women were asked about their patterns of ATS use and the settings in which it occurred, as well as motivations for using ATS, perceived negative consequences of use and experiences attempting to abstain or reduce consumption. The mean age of participants was 25 years, just over one quarter (26.7 %) were currently single, and two thirds (63.4 %) had one or more children. The majority of women (86.6 %) had completed five or fewer years of schooling, and most (66.7 %) lived in private rental accommodation, typically single rooms, at the time of interview. Primary work venues at the time of interview included karaoke bars (46.7 %), brothels (16.7 %) and parks and streets (23.3 %).

### ATS use in the context of sex work

Participants highlighted the salience of ATS in the context of sex and entertainment work. Women reported using ATS for occupational performance—to stay awake longer and to work more hours, enabling them to see more clients.

*[M]ost importantly if we don’t use it, it makes us rather sleepy. We could not stand [solicit clients] feeling sleepy until dawn as we could earn no money* (Vanna, 32 year-old FESW, Battambang).

*For those who work in entertainment services, when we smoke it, we can earn money. How can we earn money? We can stand till dawn. Stand all night till dawn. It is up to us. When the substances are in our body we cannot sleep so we stand earning money* (Rumdoul, 23 year-old FESW, Phnom Penh).

*The reason is that I first separated from my husband and I don’t have money to support my children so I decided to do so because I don’t have energy to work at night then I have to use it* (Channa, 20 year-old FESW, Phnom Penh).

ATS were valued for their pharmacological properties, primarily the inducement of insomnia and increased energy or strength. Outside the occupational setting and without this functional purpose, women indicated that they were less likely to use ATS.

*I use it a little not to feel sleepy so that I can earn money to support my children. Most importantly, I am happy as I sleep with my children at home. I ate rice. I went to the market to buy clothes for my children. This makes me happy* (Sopha, 32 year-old FESW Battambang).

*Sometimes we don’t have enough power so no matter how much we use it [yama] we must sleep. At that time we could not have more sex, so we don’t use it when we could not have more sex because we would waste our money. We just sleep to gain power and eat fully and wait until the time we earn money and we use it then we have power* (Chorphum, 30 year-old FESW, Banteay Meanchey).

While insomnia, increased energy and endurance were not seen as functional outside the workplace, within the context of sex work functionality, the performance-enhancing aspects of ATS were the most commonly mentioned motivations for use.

*[S]ometimes when we don’t smoke it, we can’t earn money. When we smoke it, we don’t feel sleepy, then we can work… [W]e just want to smoke it to earn money* (Chorvy, 22 year-old FESW, Phnom Penh).

*I used it not to feel sleepy as I sought to work overtime only to earn more money* (Mala, 20 year-old FESW, Siem Reap*).*

A minority of women indicated that using ATS also made them feel happy or helped to alleviate boredom and hunger.

*I work at night and when I don’t use it, I feel tired and physically uneasy and drowsy if I don’t use it. When I use it, I don’t feel drowsy and I have power to work. [Is there any other reason why you like to use ice?] I obviously feel happy when I use it. And don’t feel bored at all. When I don’t use it, I feel frustrated and bored. And feel as if I have no energy in my body* (Sonary, 20 year-old FESW, Kandal).

*[Why do you like to use the ice?] Oh, when we smoke it, we feel strange. And feel happy and delicious. Delicious and happy. We feel that we are not hungry for rice* (Rumdoul, 23 year-old FESW, Phnom Penh).

However, most women reported using ATS not because they were seeking pleasure or avoidance, but rather out of economic necessity engendered by extreme poverty and the occupational realities of sex work.

*[ATS] helps me to feel not sleepy … when a customer pays to have sex with us all night and the customer is kind he allows us to leave him early, so we can come back … If the customer allows us to leave them early, we can go find one more customer, so we can get money to buy something to eat for tomorrow* (Chorphum, 30 year-old FESW, Banteay Meanchey).

For the women in our study, the pharmacological properties of ATS rendered them functional in the context of sex work. Indeed the physiological effects of ATS reported here, including insomnia and appetite suppression, enabled women to work long hours, see more customers and avoid the need for rest or food. FESW perceived the impact of ATS to be not only physiologically and hence economically functional but also largely positive, helping them to ‘be happy’ and forget about their problems. While previous research also indicates that some sex workers use drugs to cope with their work [25], women in our study described ATS as a central, normalized, and even obligatory aspect of their work practice.

#### ATS and clients and managers

Some women also described how ATS use made them feel less shy or inhibited with clients, making them more comfortable and amicable.

*The reason why I started to use it is that I could not stand being sleepy and when we receive the guests, we are not shy with them. In short, we dare to go there, no matter how many times, we dare to go* (Ratana, 20 year-old FESW, Phnom Penh).

Thus, in addition to being a source of physical energy or ‘power’, ATS use is also functional for women on a psychological level, helping to lower inhibitions, curb shyness and facilitate interactions with clients.

*[I]f I use it when I go to work and the guests call me, I feel like going with the guests. If I don’t use it, when the guests call me, I don’t feel like going and get angry … I didn’t use it much before but now I start to use it so often. Since I began my work six months ago I use it much. I think that I have that kind of job [where I must take ATS] to feel like wanting to go with the guests* (Sonary, 20 year-old FESW, Kandal).

*[I]t makes us not afraid at all, not shy at all* (Votey, 22 year-old FESW, Siem Reap).

Several women highlighted the role of clients or guests in demanding or encouraging women to use ATS and by expressing a preference for FESW who used ATS.

*We need it so that we can work for long hours and don’t feel too sleepy … Guests also like to use it. But if we don’t know how to use it they don’t like us* (Haratey, 24 year-old FESW, Phnom Penh).

*We have to use it. Let’s say, the guests say we have to use it to have the feeling, and we use it. It feels like this and that. We have to use it. I would say we do such work, we have to take risk. If we don’t, we won’t get money from them. So we have to take risks* (Srey Neang, 27 year-old FESW, Phnom Penh).

*In short, they let us use it so that they can have long sexual intercourse with us* (Ratana, 20 year-old FESW, Phnom Penh).

Clients or guests facilitated women’s use of ATS by affording them drugs, reinforcing their use, and subjecting them to extended sexual transactions (see ‘Negative consequences of ATS use below).

Employers and managers, as well as colleagues and friends, also exerted pressure on women to use ATS.

*I used yama because I had my boss’ [support] and I just used it without thinking about anything. At that time I used it without thinking about anything because I had house, space for sleeping and meals to eat. Although I did not generate any income, I still had rice to eat and place to sleep, so I did not care about anything. Therefore, no money remained even if I calculated it once per month. [Our money] was just enough to pay back for drugs but at that time we were not allowed to go out to buy drugs freely. The boss bought it for us* (Chorphum, 30 year-old FESW, Banteay Meanchey).

*[I]t is a must to do so at work and I have to go with guests. If I don’t go with guests, I don’t want to use it either, even when my friends persuade me I don’t [want to use]* (Channa, 20 year-old FESW, Phnom Penh).

In particular, our data suggest that ATS use by FESW may also be functional for male managers and bosses. Women who used ATS worked longer hours and saw more clients, potentially earning additional income for managers. In addition, many clients expressed a preference for FESW who used ATS with them, allowing managers of ATS-using FESW to meet this demand. However, a minority of women indicated that some managers and bosses discouraged women from using ATS.

*They said they don’t allow me to work … My boss wanted me not to smoke it. My boss told me, “If you still smoke it, I no longer allow you to work”. I was reducing it a little bit then. My boss said if I continued to reduce or stop it, I would get prettier soon* (Ratana, 20 year-old FESW, Phnom Penh).

#### ATS as a moderator of occupational alcohol consumption

Previous research has shown that alcohol consumption is related to sex work occupational settings, with women working in entertainment venues more likely to drink more alcohol than brothel or street-based FESW [26]. Several women who worked in entertainment venues drew attention to this further dimension of occupational ATS use. These women indicated that they and others used ATS in order to moderate or reduce the effects of occupational alcohol consumption.

*When people [first] called me to use it [yama] I thought I did not want to touch it but at that time I was forced to do so because I had to drink heavily so I thought that I could control my feeling as people said that when using it, [yama] our feeling would be clear and weaken the effect of alcohol. They made our feeling clear so we thought that it was a good thing if we were sober* (Viriya, 23 year-old FESW, Banteay Meanchey).

*When I get drunk, I also want it. It’s like a diluting drug. No matter how many cases of alcohol. I drink with the guests until I get so drunk but when I use it I don’t get drunk anymore. I no longer get drunk* (Ratana, 20 year-old FESW, Phnom Penh).

*It [ATS] can weaken the wine’s effects when we are drunk* (Morokoth, 18 year-old FESW, Kandal).

*I work at night and I need to drink [alcohol]. Whenever I drank it [ATS] it could reduce drowsiness and we could drink much [alcohol]* (Manavy, 25 year-old FESW, Kandal).

Our previous research found that almost half (44.3 %) of young (15–29 years) FESW reported being drunk on five or more days in the last month [14]. The high prevalence of alcohol use among Cambodian FESW is of concern, and currently, no interventions target alcohol use in this population. The data presented above suggest that some FESW use ATS in order to mitigate the effects of alcohol consumption that is an inherent part of their employment in entertainment venues. While using ATS to ‘dilute’ the effects of alcohol represents an attempt by women to control or reduce risk, it exposes women to a different and heightened set of risks. This combination of individual and structural risk factors presents a complex jeopardy for FESW—not only to their health but also their livelihood—and a very challenging situation for the development of interventions aimed at reducing risks.

### Negative consequences of ATS use

Women also reported negative consequences of ATS use, including undesirable and adverse impacts on physical and mental health.

*We would lose our intention and energy and anything we want, may lose it accordingly as the drugs could make us unable to sleep much. As we could not eat much every day, our health would deteriorate accordingly* (Leahkhena, 29 year-old FESW, Battambang).

*[W]hen it loses its influence [effects wear off], in the morning we feel physically uneasy and we are exhausted. It is just like a person being beaten with hundreds of sticks* (Serey, 19 year-old FESW, Kandal).

*I don’t want to use drugs. First it makes me addicted. Second it troubles my brain’s nerves. I previously remembered things well but I [now] have problems in my brain* (Haratey, 24 year-old FESW, Phnom Penh).

*If I smoke it on any day and have any feeling of hatred for anyone, I provoke them at once. I provoke them, want to beat them, want to kill them. That’s very bad. If I feel like doing anything, I do it* (Ratana, 20 year-old FESW, Phnom Penh*).*

Several women also described the impact on their physical appearance as a negative consequence of ATS use.

*I always wonder and could not eat rice. I have hollow cheeks and I was not like this before. I was very pretty and I felt so regretful …You can ask Smart Girl how pretty was I before. Yes, that’s why I said I felt sorry for my body so much. That’s why I said using drugs would let us down, humiliate us, kill us, make us suffer and also make us have no rice to eat* (Chakriya, 25 year-old FESW, Battambang).

*When looking around my house, there are a lot of drugs users. Talking about their appearance, they look ugly. Their beauty gets worse. So I think that when I work and I look ugly, they won’t choose me. They won’t love me… But I have to reduce it to some extent not to let them say that I look too bad. When we use it, what a face [laughter]. It looks bad. It makes our eyes look pale, our face look angular in shape and without full flesh and blood* (Srey Neang, 27 year-old FESW, Phnom Penh).

However, in the face of limited income-generating options, perceptions of negative health impacts rarely provided sufficient motivation for women to reduce their use.

*Last month I used it almost every day per week. I went there almost every day. I turned black, pale, thin and my clothes are all loose and when I go to work, they no longer accept me for work. They said I have a very ugly face. When we suffer from its effects, we think that we are beautiful. Others see us as a crazy person. I used powder to make up my face then they said I was crazy* (Ratana, 20 year-old FESW, Phnom Penh).

ATS, particularly methamphetamine use, has been associated with ‘hypersexuality’, including sexual marathons, among MSM [3, 27] and heterosexual methamphetamine users [6]. Consistent with this literature, women in the current study reported that ATS use by clients often delayed ejaculation, resulting in prolonged sexual activity.

*[G]uests like to use it. They like to use it so that they do not ejaculate so fast. It makes them do it for a long time. When they sleep with us, they say that they want it to be long … and they say that when they use it, it makes them happy and sexually aroused* (Haratey, 24 year-old FESW, Phnom Penh).

*So without drugs I cannot have sex either. So when I used it [ATS] and I had sex with the customers for half an hour, I could endure it* (Chorphum, 30 year-old FESW, Banteay Meanchey).

Our data are consistent with previous studies which have documented associations between ATS use and increased sexual risk [5, 10, 28]. However, the association between ATS use and sexual risk is often attributed to disinhibition and the impact of the drug on sexual decision-making [4]. In the current study, women reported that their ATS use helped them cope with protracted sexual activity resulting from clients’ use. There is potential for increased HIV risk associated with tissue damage in this context [29]. This kind of aggravated ‘occupational’ risk also points to the need for interventions designed to change the drug use and sexual behaviour of male clients.

### Reducing or abstaining from ATS use

In order to inform development of the CCT intervention, we also explored women’s motivations for, and experiences of, reducing or abstaining from ATS use. Few women believed that they were physiologically dependent on ATS.

*It is very easy for us to stop when the effect of the addictive substances is weakened. We feel sleepy. We sleep only and we are hungry. We start eating for a period of one day and within a period of two days the effect of the addictive substances is weakened. Within a period of three days it will be gone* (Chakriya, 25 year-old FESW, Battambang).

However, not using ATS meant reduced functionality in their occupational role as sex workers.

*I can reduce or stop the ice because it’s not so addictive if we try. But because we work at night, we can’t do it yet. I think that if I get better off [have more income] I may be able to stop it* (Channa, 20 year-old FESW, Phnom Penh).

This functional purpose dominated other considerations and, as noted above, was the primary driver of ATS use among the women interviewed.

*I had nothing for our children to eat daily so I needed it. I stayed until three or two and a half [am] to earn money for raising my children on a daily basis … I completely quit it myself when I did not go to work. Although I saw my friends like using it or they brought it to me, I did not use it* (Saran, 22 year-old FESW, Battambang).

For most women, poverty, low literacy and limited skills meant that sex work was often the only occupation open to them. However, the functional role of ATS in helping women to stay awake and work long hours meant that it was difficult for women to quit or reduce their use while working as sex workers. As the accounts below illustrate, many women were able to stop or reduce ATS use during periods when they were not working—either going to their home town to visit family or following childbirth.

*Yes, in the past I used to stop. I used to stop it … Stay with friends, relatives who may not know this. We stay normally. We forget and we eat only rice and sleep… [And why later on did you use it again?] [I]t’s just that we come to stay here and when it’s time to work we use* (Daraneat, 30 year-old FESW, Banteay Meanchey).

*To abstain from it, I went to do farming … I committed myself to transporting seedlings alone and tried to ignore it when I wanted to use it. I abstained from drugs for a short period … Unfortunately, my mom fell sick. I decide to come back to work in Poipet and then my mom came to live with me. I went to rent a house outside for two months and I did not use drugs for those two months. It was because my mom was with me. When my mom left, I went to work and my friends asked me to smoke again* (Phany, 19 year-old FESW, Banteay Meanchey).

For the women in our study, ATS use was deeply enmeshed with sex work and embedded in the occupational role. Among those who managed to quit ATS and/or sex work, recurrent financial problems often precipitated a return to work and a subsequent relapse to drug use.

*[I] returned to use it [ATS] when my child got sick. The elder one was at a hospital and we spent all the money. We did not have money then borrowed interest-bearing loan from others to send my child to hospital and needed to meet guests [return to sex work] to earn money to repay* (Haratey, 24 year-old FESW, Phnom Penh).

*I started using it since I was 16 years old. When I was 23 years old I got married and I stopped going … I stopped [working] for fifteen years and I quit forever. [And when did you start using again?] Since I was separated from my husband I started using it since June 2013. … Then I had to work at karaoke. Then I also got drunk [at work]. And I could not stand feeling sleepy then my friends said that if I used it before I could use it again. But I thought that I did not want to use it. I said to myself that I would not do it again. But I could not stand it. I felt sleepy and if I did not do this, I would not use it. We try not to feel sleepy but we could no longer be able to earn money to support our children* (Sopha, 32 year-old FESW, Battambang).

These data indicate a need for interventions designed to provide women with both short-term incentives for behaviour change and longer-term alternative methods of income generation. Given the occupational salience of ATS use, CCT interventions offer the potential to incentivise women who wish to reduce their use by rewarding them for negative urine toxicology screens, as well as compensating them for fewer hours worked.

## Conclusions

Most participants in our study had little formal education and few options for income generation beyond sex work. The key driver of ATS use in this group was increased functionality, including physical stamina, and coping with the multiple demands of work comprising long working hours, multiple clients and extended sexual transactions. While our findings are consistent with previous research linking ATS use to heightened sexual risk via unprotected and/or prolonged sex, for women in the current study, the negative consequences of ATS use were outweighed by perceived functional benefits. This was true even when these benefits correlated with increased sexual risk. Even where women were aware that ATS use could affect their health and income-generating capacity in the longer term, occupational functionality in terms of increased stamina and performance at work was at the core of their narrative accounts and remained the primary motivation for use. ATS use was a central, and often acceptable, part of their work.

Our results are consistent with studies which have identified occupational functionality as a driver of drug use in other settings including among fisherman [30], truck drivers [31, 32], military personnel [33], artists, musicians and writers [34, 35] and, most recently, the academically-oriented use of cognitive-enhancing drugs in higher education [36, 37]. In Asia, a study examining the social and economic functions of methamphetamine use among Thai youth found that more than a third reported using the drug prior to job-related activities [38]. Similarly, research exploring methamphetamine use among marginalised young male vendors in a Philippine port found that methamphetamine use was associated with increased strength and confidence, disinhibition and insomnia [39], and, importantly, that methamphetamine was viewed as a ‘pampagilas’ or performance enhancer [39]. Most recently, Choo [40] has shown how injecting drug use represented an adaptive response by Malay fisherman to declines in traditional fisheries and increases in commercial vessels and foreign labour. Within this setting, while drug use increased exposure to HIV, it also provided Malay fisherman with a way of enhancing occupational functioning [40]. Similarly, we found that while ATS use by Cambodian FESW increased their risk of HIV, it enhanced their occupational functioning, enabling them to work longer hours, see more clients and forgo food and rest.

Elsewhere, the presence of social, family and occupational supports and obligations has been associated with the controlled use of drugs, including heroin and cocaine [41–43]. Our data suggest that the functionality of ATS use and its normalization in this setting may account, in part, for the high prevalence of ATS use among FESW in Cambodia [14]. Economic pressures on women were often intensified by obligations to support partners and families—the latter being particularly strong in Cambodian culture. Participants gave us insight into narratives of ATS use as functional and as facilitating a sense of power and agency. Such narratives contrast with those found in the literature on MSM, where ATS use has been situated almost exclusively within discourses of enhanced sexual performance [44, 45] and sexual risk [4]. Our results suggest that some of the negative effects of ATS use, including the risk of HIV, may be mediated by perceived occupational functionality in sex and entertainment work settings.

Our analysis has implications for HIV prevention efforts in Cambodia and elsewhere. Current approaches focus on empowering individual FESW to negotiate condom use. However, these strategies often fail to appreciate the complexities of commercial sex transactions where drug use, including ATS use, is involved. Our data highlight the need for a harm-reduction framework that acknowledges not only the intersection of drug use and sex work [46], but also the existence of strong occupational incentives to use ATS. ATS use by FESW is clearly also functional for male clients and some bosses or managers of entertainment venues. Indeed, the prevalence of ATS use among FESW, supported by both clients and ‘bosses’ or employers, fosters a culture of drug use within the Cambodian sex and entertainment industry. The acceptance and, in some situations, active encouragement of ATS use suggests a need to engage both venue owners and employers, as well as clients, in harm-reduction efforts.

An important challenge for Cambodia at this stage of its HIV epidemic—when it aims to eliminate new HIV infections [47]—is to target interventions where they will have greatest impact. Recent mathematical modelling based on data from Africa indicates that sex workers with the highest rates of partner change have a disproportionately high impact on transmission [48]. Our results suggest that targeting FESW who use ATS is likely to yield prevention benefits in terms of identifying an active sub-population who see more clients, report higher levels of unprotected sex, and are greater risk of HIV and other sexually transmitted infections.

Within this context, there is clearly a need to increase awareness among FESW of the risks associated with ATS use, including the risk of HIV/STI infection associated with unprotected sex and potential tissue damage as a result of extended sexual activity and, in the longer term, the potential for dependence, mental health issues, physical degeneration and diminished capacity for income generation. Our data also highlight the role of alcohol in mediating the effects of ATS, suggesting that interventions also need to address the occupational use of alcohol by FESW working in entertainment venues. And while behavioural interventions designed to reduce HIV risk among methamphetamine users have been developed, most of these interventions target MSM in developed countries [49] rather than FESW in resource-poor settings. Our data indicate the promise of interventions based on behavioural economics, including CCT programmes designed to prevent HIV and STI by incentivising FESW to reduce their drug use.

FESW in Cambodia harness the pharmacological properties of ATS to meet the physiological demands of sex work in a context of limited economic opportunities. In this setting, where up to one in four young FESWs are infected with HIV, there is an urgent need to both provide women with options for income generation that do not risk their health, to better regulate the conditions of sex work to promote safer working environments [50, 51], and to afford sex workers the same benefits and protections afforded other workers [52]. Structural and economic interventions, combined with increased awareness and enforcement of sex worker’s rights, are necessary to facilitate harm reduction and occupational health and work safety within the Cambodian sex and entertainment industry.
